# Alisol B 23-Acetate Inhibits IgE/Ag-Mediated Mast Cell Activation and Allergic Reaction

**DOI:** 10.3390/ijms19124092

**Published:** 2018-12-18

**Authors:** Chen Shao, Bingjie Fu, Ning Ji, Shunli Pan, Xiaoxia Zhao, Zhe Zhang, Yuling Qiu, Ran Wang, Meihua Jin, Ke Wen, Dexin Kong

**Affiliations:** 1 Tianjin Key Laboratory on Technologies Enabling Development of Clinical Therapeutics and Diagnostics, School of Pharmacy, Tianjin Medical University, Tianjin 300070, China; 15840941135@163.com (C.S.); fubingjie@tmu.edu.cn (B.F.); ningji@tmu.edu.cn (N.J.); panshunli@tmu.edu.cn (S.P.); zhaoxiaoxia@tmu.edu.cn (X.Z.); zhangzhe@tmu.edu.cn (Z.Z.); qiuyuling@tmu.edu.cn (Y.Q.); wangran@tmu.edu.cn (R.W.); 2 Department of Pharmacology, College of Basic Medical Sciences, Tianjin Medical University, Tianjin 300070, China; 3 Research Center of Basic Medical Sciences, Tianjin Medical University, Tianjin 300070, China

**Keywords:** AB23A, mast cell, IgE, PCA

## Abstract

Alisol B 23-acetate (AB23A), a natural triterpenoid, has been reported to exert hepatoprotective and antitumor activities. Aiming to investigate the anti-inflammatory activity, this study examined the effect of AB23A on mast cells and allergic reaction. AB23A inhibited the degranulation of mast cells stimulated by immunoglobulin E/antigen (IgE/Ag), and also decreased the synthesis of leukotriene C_4_ (LTC_4_), production of interlukin-6 (IL-6), and expression of cyclooxygenase-2 (COX-2) in a concentration-dependent manner with no significant cytotoxicity in bone marrow-derived mast cells (BMMCs). AB23A inhibited spleen tyrosine kinase (Syk) and the downstream signaling molecules including phospholipase Cγ (PLCγ), serine-threonine protein kinase/inhibitor of nuclear factor kappa-B kinase/nuclear factor kappa-B (Akt/IKK/NF-κB), and mitogen-activated protein kinases/cytosolic phospholipase A_2_ (MAPK/cPLA_2_). Furthermore, AB23A blocked mobilization of Ca^2+^. Similar results were obtained in other mast cell lines Rat basophilic leukemia (RBL)-2H3 cells and a human mast cell line (HMC-1). In addition, AB23A attenuated allergic responses in an acute allergy animal model, passive cutaneous anaphylaxis (PCA). Taken together, this study suggests that AB23A inhibits the activation of mast cells and ameliorates allergic reaction, and may become a lead compound for the treatment of mast cell-mediated allergic diseases.

## 1. Introduction

Mast cells play an important role in the initiation and propagation of various inflammatory disorders such as asthma, atopic dermatitis, arthritis, etc [1]. Mast cells express large numbers of the high-affinity immunoglobulin E (IgE) receptor FcεRI. The Antigen (Ag) cross-linking of Ag-specific IgE bound to FcεRI induces aggregation of FcεRI which activates multiple signaling pathways, leading to secretion of inflammatory mediators implicated in allergic or inflammatory reactions, such as histamine, leukotrienes (LTs), and cytokines [2,3].

Interaction of a multivalent antigen with FcεRI induces the activation of the spleen tyrosine kinase (Syk), and phosphorylation of linker for activated T cell (LAT). These proteins then serve as scaffolds for multi-molecular signaling complexes for the binding of cytosolic adapter molecules such as Gads, Grb_2_, and SLP76, guanosine triphosphate exchangers including Sos and Vav1, and the signaling enzymes phospholipase Cγ1 (PLCγ1) and PLCγ2 [4]. Activated PLCγ positively regulates protein kinase C (PKC) and liberates intracellular Ca^2+^ [5]. Activation of mitogen-activated protein kinases (MAPKs) enhances the production of arachidonic acid (AA) and its metabolites [5]. These signals lead to mast cell degranulation, eicosanoid generation, and cytokine production.

Alisol B 23-acetate (AB23A), a natural triterpenoid from a Chinese medicinal herb, Alismatis Rhizoma, has multiple physiological activities including anticancer, hepatoprotective, and antibacterial activities [6,7,8]. However, the anti-inflammatory effect of AB23A in IgE/Ag-induced activation of bone marrow-derived mast cells (BMMCs) has not yet been reported. With the aim of evaluating the anti-inflammatory effect of AB23A and elucidating the related mechanism, we recently examined the effect of AB23A on the release of histamine, synthesis of LTC_4_, generation of interleukin (IL)-6, and expression of cyclooxygenase (COX)-2 in IgE/Ag-stimulated mouse BMMCs. To investigate the possible mechanism involved, the effect on molecules in the FcεRI signaling pathway including nuclear factor kappa B (NF-κB), mitogen-activated protein kinases (MAPKs), Syk, Src-family kinases Fyn and Lyn, PLCγ, serine-threonine protein kinase C (Akt), cytosolic phospholipase A_2_ (cPLA_2_), and cytosolic Ca^2+^ mobilization was examined. In addition, we used other mast cell Rat basophilic leukemia (RBL-2H3) and human mast cell line (HMC-1) to demonstrate the anti-inflammatory effects of AB23A. Finally, we evaluated the anti-allergic effect of AB23A on AN IgE/Ag-mediated passive cutaneous anaphylaxis (PCA) reaction in vivo.

## 2. Results

### 2.1. AB23A Suppresses Histamine Release and Ca^2+^ Mobilization in IgE/Ag-Stimulated BMMCs by Inhibiting PLCγ Phosphorylation

**Figure 1:**
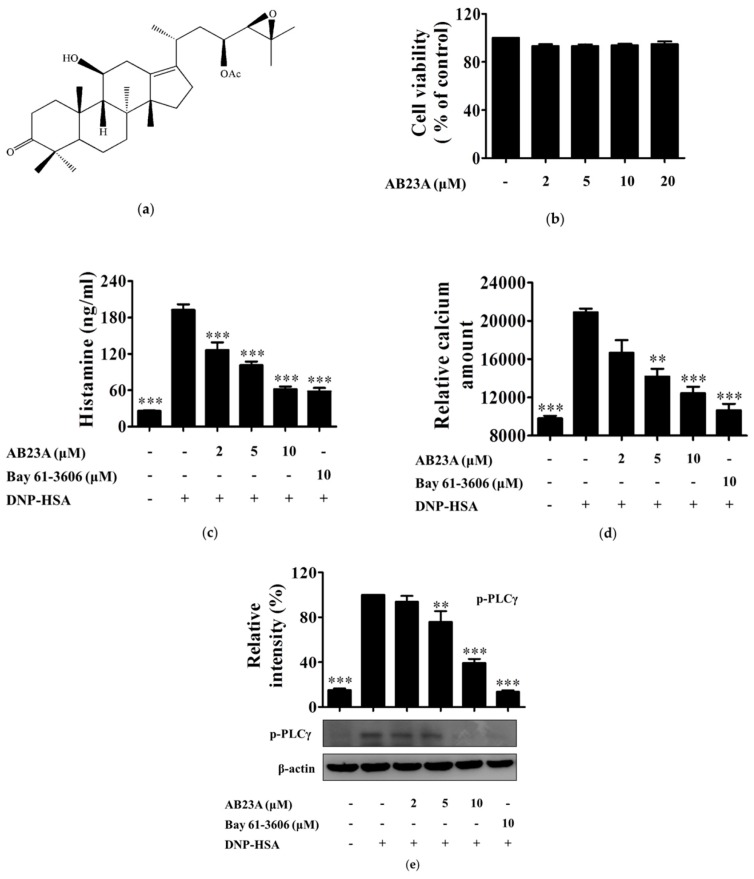
AB23A inhibits histamine release and Ca^2+^ mobilization by phospholipase Cγ (PLCγ) phosphorylation in immunoglobulin E/antigen (IgE/Ag)-stimulated bone marrow-derived mast cells (BMMCs). (**a**) Chemical structure of AB23A; (**b**) incubation of BMMCs with various concentrations (2, 5, 10, and 20 μM) of AB23A for 8 h. Cells cytotoxicity was determined by MTT assay; (**c**) IgE-sensitized BMMCs were pre-treated with AB23A or Bay 61-3606 for 1 h, and then stimulated with DNP-HSA for 15 min. The amount of histamine released into the culture media was measured by ELISA; (**d**) IgE-sensitized BMMCs were pre-incubated with FluoForte TM dye-loading solution for 1 h, and then treated with AB23A or Bay 61-3606 for 1 h. The fluorescence was measured after stimulation with DNP-HSA for 5 min; (**e**) IgE-sensitized BMMCs were stimulated with DNP-HSA for 15 min after being pre-treated with AB23A or Bay 61-3606 for 1 h. The cell lysates were collected and immunoblotted with antibody for phospho-PLCγ, the relative ratios of p-PLCγ was determined by analyzing immunoblot band intensities. The data show the mean ±SEM of three independent experiments. Analysis of variance (ANOVA), *p* < 0.0001, post hoc ** *p* < 0.01, and *** *p* < 0.001, compared with the BMMCs stimulated with IgE/Ag in the absence of AB23A.

We initially used a 3-(4,5-dimethyl-2-thiazolyl)-2,5-diphenyl-2H-tetrazolium bromide (MTT) assay to determine the cytotoxicity of AB23A, see Figure 1a, against BMMCs. As a result, treatment with 2, 5, 10, and 20 μM of AB23A for 8 h did not show obvious cytotoxicity, see Figure 1b. Thus, concentrations of 2, 5, and 10 μM were chosen for an in vitro anti-inflammatory study. Mast cells store large amounts of histamine in their granules, which are released by degranulation in response to different stimuli [9]. Therefore, we investigated whether the AB23A inhibits histamine release in BMMCs. BMMCs were sensitized with IgE overnight, pre-treated with AB23A for 1 h, and then stimulated with anti-dinitrophenyl (DNP) IgE-human serum albumin (HSA) for 15 min. The release of histamine was measured by ELISA assay. As a result, IgE and Ag significantly induced histamine release in BMMCs (*p* < 0.001), and the induced-release of histamine was suppressed by treatment with 2, 5, and 10 μM of AB23A significantly (*p* < 0.001) and in a concentration-dependent manner, as shown in Figure 1c. Bay 61-3606, a Syk inhibitor used as a positive control, also inhibited histamine release significantly (*p* < 0.001).

FcεRI engagement induces phosphorylation of PLCγ, and production of IP_3_, resulting in the release of Ca^2+^ from endoplasmic reticulum (ER) [10]. The increase of the intracellular Ca^2+^ concentration is crucial for mast cell degranulation [11]. Thus, we next investigated the effect of AB23A on Ca^2+^ mobilization. As shown in Figure 1d, the intracellular amount of Ca^2+^ was increased by IgE and Ag stimulation. As expected, the intracellular Ca^2+^ levels were inhibited by AB23A in IgE/Ag-stimulated BMMCs, especially at concentrations of 5 (*p* < 0.01) and 10 μM (*p* < 0.001). Furthermore, the IgE/Ag-induced phosphorylation of PLCγ in BMMCs, whereas the increased phosphorylation was dose-dependently inhibited by AB23A treatment, as shown in Figure 1e.

### 2.2. AB23A Inhibits LTC_4_ Generation via Blocking the Phosphorylation of p38 and ERK and Translocation of cPLA_2_ into the Nuclear Envelope

**Figure 2:**
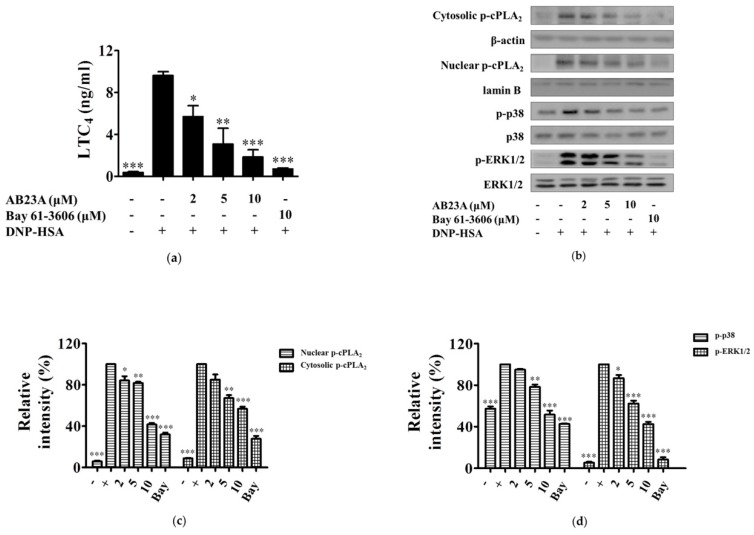
AB23A suppresses the generation of leukotriene C_4_ (LTC_4_), translocation of activated cPLA_2_, and phosphorylation of p38 and ERK1/2 mitogen-activated protein kinases (MAPKs). IgE-sensitized BMMCs were pre-incubated with AB23A or Bay 61-3606 for 1 h, and then stimulated with DNP-HSA for 15 min. (**a**) The culture medium was collected and measured by ELISA for the generation of LTC_4_; (**b**) cytosolic and nuclear fractions were immunoblotted with antibodies for phospho-cPLA_2_. Additionally, total cell lysates were immunoblotted for phosphorylated and total forms of p38 and ERK1/2; (**c**,**d**) relative ratios of cytosolic and nuclear p-cPLA_2_ proteins, p-p38 and p-ERK1/2, were measured by analyzing immunoblot band intensities. “-”: BMMCs only sensitized with IgE; “+”: IgE-sensitized BMMCs stimulated with DNP-HSA. The data show the mean ±SEM of three independent experiments. ANOVA, *p* < 0.0001, post hoc * *p* < 0.05, ** *p* < 0.01, and *** *p* < 0.001, compared with the BMMCs stimulated with IgE/Ag in the absence of AB23A.

LTs as a pro-inflammatory factor can cause increased endothelial permeability, contraction of vascular smooth muscle, and enhanced mucus secretion [12]. As shown in Figure 2a, the synthesis of LTC_4_ after stimulation with IgE/Ag was significantly increased and was about 9.6-times greater over that of the non-treated group. AB23A dose-dependently inhibited LTC_4_ generation in IgE/Ag-stimulated BMMCs, especially at a concentration of 10 μM (*p* < 0.001). LTs are derived via the 5-lipoxygenase (5-LO) pathway of arachidonic acid (AA) metabolism [13]. Under resting conditions, cPLA_2_ and 5-LO reside in the cytoplasm. The increase of intracellular Ca^2+^ leads to the translocation of 5-LO to the nuclear membrane, where it associates with the scaffold protein 5-lipoxygenase-activating protein (FLAP). Meanwhile, cPLA_2_ translocates from the cytosol to the nuclear membrane. These make up the core of the LT biosynthetic complex [13]. To determine whether AB23A modulates the translocation of cPLA_2_, BMMCs were treated with AB23A, and the cytosolic and nuclear phosphorylated cPLA_2_ were measured. The phosphorylation of cPLA_2_ in BMMCs was significantly increased by the IgE/Ag challenge. However, the phosphorylation, as well as the translocation of cPLA_2_, was strongly suppressed by AB23A in activated BMMCs, see Figure 2b,c.

Phosphorylation of cPLA_2_ precedes the increase of intracellular Ca^2+^ to fully activate the enzyme and mobilize AA for eicosanoid production, and cPLA_2_ activation correlates with the activation of p38 and ERK [14]. In order to investigate whether AB23A affects the up-stream signal of cPLA_2_, next, we measured the effect of AB23A on the phosphorylation of ERK1/2 and p38 MAPK. The Ag challenge for 15 min increased the phosphorylation of p38 and ERK1/2 in BMMCs, which was down-regulated by AB23A, see Figure 2b,d.

### 2.3. AB23A Decreases IL-6 and COX-2 Expression by Suppressing the Akt/IKK/NF-κB Pathway

**Figure 3:**
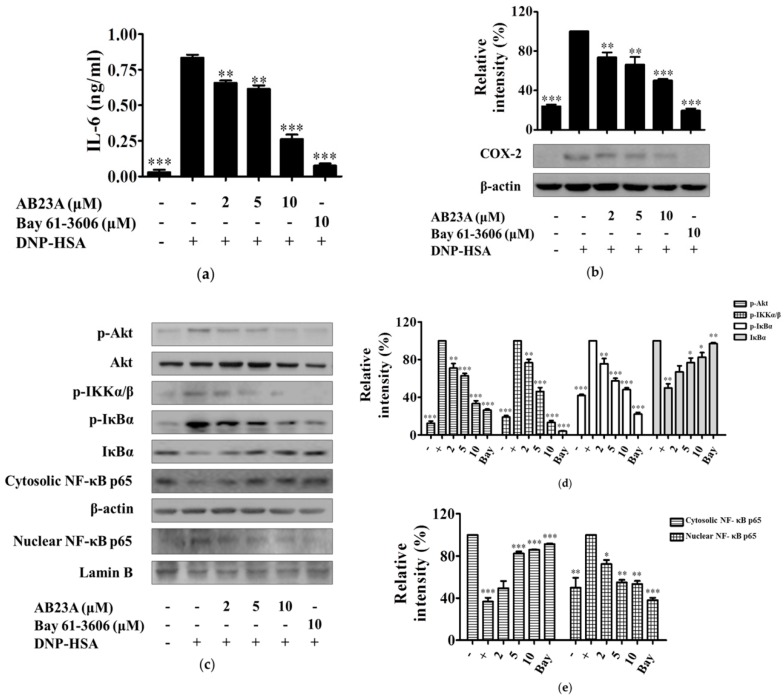
AB23A inhibits the production of IL-6 and the expression of COX-2 through part of the Akt/IκB/NF-κB pathway. (**a**) IgE-sensitized BMMCs were pre-treated with AB23A or Bay 61-3606 for 1 h and stimulated with DNP-HSA for 6 h. IL-6 were measured by ELISA. (**b**) IgE-sensitized BMMCs were pre-incubated with aspirin for 2 h to eliminate the activity of COX-1. After washing, the BMMCs were treated for 1 h with the AB23A or Bay 61-3606 and then stimulated with DNP-HSA for another 7 h. Cell lysates were immunoblotted with the antibody for COX-2. The relative ratio of COX-2 protein was measured by analyzing the immunoblot band intensity. (**c**) IgE-sensitized BMMCs were pre-incubated with AB23A or Bay 61-3606 for 1 h and then stimulated with DNP-HSA for 15 min. Cell lysates were immunoblotted for p-Akt/Akt, p-IKKα/β, p-IκBα/IκBα; cytosolic and nuclear NF-κB p65. Relative ratios of the p-Akt, p-IKKα/β, p-IκBα, IκBα (**d**), and cytosolic and nuclear NF-κB p65 (**e**) proteins were determined by analyzing immunoblot band intensities. “-”: BMMCs sensitized with only IgE; “+”: IgE-sensitized BMMCs were stimulated with DNP-HSA in the absence of AB23A. The data show the mean ±SEM of three independent experiments. ANOVA, *p* < 0.0001, *p* < 0.01 (**d**: IκBα), *p* < 0.001 (**e**: nuclear NF-κB p65), post hoc * *p* < 0.05, ** *p* < 0.01, and *** *p* < 0.001, compared with the BMMCs stimulated with IgE/Ag in the absence of AB23A.

IL-6 is a pro-inflammatory cytokine that plays an important role in inflammatory diseases such as rheumatoid arthritis, systemic juvenile arthritis, psoriasis, and ankylosing spondylitis [15]. Without Ag stimulation, IL-6 was barely produced in BMMCs. However, after being activated by IgE/Ag, the level of IL-6 increased significantly (*p* < 0.001). Further treatment with AB23A markedly reduced IL-6 production in a dose-dependent manner, see Figure 3a. 

COX-2 is well-known to play a key role in the pathogenesis of inflammatory diseases. This enzyme is upregulated in inflammatory situations and responsible for the production of prostaglandins (PGs) from AA [16]. In order to detect the inhibitory effect of AB23A on the expression of COX-2, IgE-sensitized BMMCs were treated with aspirin for 2 h to abolish preexisting COX-1, and then stimulated with DNP-HSA for 7 h in the presence or absence of AB23A. As a result, the expression of COX-2 in BMMCs was increased by IgE/Ag stimulation, and AB23A dose-dependently inhibited the expression in a concentration-dependent manner, see Figure 3b. 

The transcription factor, NF-κB, is a central regulator of inflammation and modulates several biological processes. In normal resting cells, p65/p50 heterodimers are maintained in the cytoplasm as an inactive form by inhibitor of NF-κB (IκB). During activation of NF-κB, NF-κB was liberated from the IκB and rapidly translocated to the nucleus to transcript various inflammatory mediators, such as IL-6 and COX-2 [17]. IκB is phosphorylated by IKK and then ubiquitinated [17]. IκBα is phosphorylated by the IKK complex, and IKKα/β are catalytic subunits of IKK complex [18]. As shown in Figure 3c, NF-κB p65 translocated from the cytosol into the nucleus in IgE/Ag-stimulated BMMCs, with p65 in the cytosol decreasing and p65 in the nucleus increasing after stimulation with IgE/Ag. This translocation was inhibited by AB23A treatment, see Figure 3c,e. Whereas AB23A inhibited phosphorylation of IKKα/β and phosphorylation and degradation of IkBα, see Figure 3c,d. In addition, AB23A also inhibited the IgE/Ag-induced phosphorylation of Akt in BMMCs, see Figure 3c,d.

### 2.4. AB23A Inhibits Syk Phosphorylation Independent of Lyn and Fyn 

**Figure 4:**
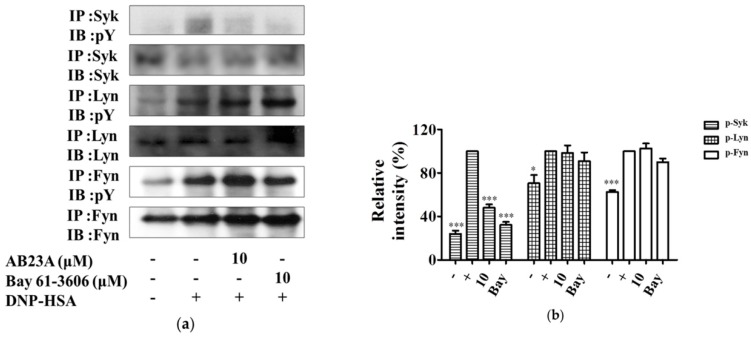
AB23A suppresses the Syk-associated pathway. (**a**) IgE-sensitized BMMCs were pre-incubated with AB23A or Bay 61-3606 for 1 h and then stimulated with DNP-HSA for 5 min. Cell lysates were subjected to immunoprecipitation and immunoblot analysis for the phosphorylated forms of Syk, Lyn, and Fyn. (**b**) The relative ratios of Syk, Lyn, and Fyn proteins were determined by analyzing immunoblot band intensities. The data show the mean ±SEM of three independent experiments. ANOVA, *p* < 0.0001 (p-Syk), *p* < 0.05 (p-Lyn), *p* < 0.001 (p-Fyn), post hoc * *p* < 0.05 and *** *p* < 0.001, compared with the cells stimulated with IgE/Ag in the absence of AB23A.

FcεRI requires the recruitment of the Src family tyrosine kinases (Lyn and Fyn) and Syk to control the early receptor-proximal signaling events [19]. Syk is essential for the propagation of signals in mast cells, following the binding of the phosphorylated immunoreceptor tyrosine-based activation motifs (ITAMs) to FcεRIγ [19]. Upon Syk activation, various adaptor molecules such as the linker for activated T cell (LAT) and Grb_2_ and signaling enzymes, PLCγ1 and PLCγ2, were activated [19]. Because AB23A inhibited activation of NF-κB and phosphorylation of MAPKs and PLCγ, we determined whether these suppressions were dependent on activation of Syk, Lyn, and Fyn. Stimulation of IgE and DNP-HSA caused an increase in phosphorylation of Syk, Lyn, and Fyn. The enhanced phosphorylation of Syk was inhibited by AB23A treatment. However, the phosphorylation of Lyn and Fyn was not changed after AB23A treatment, see Figure 4a,b. As expected, the Syk inhibitor, Bay 61-3606 also inhibited Syk phosphorylation, without affecting Lyn and Fyn. These results indicate that AB23A inhibits phosphorylation of Syk, which might contribute to its inhibitory effect on the activation of BMMCs.

### 2.5. AB23A Inhibits the Activation Signal of RBL-2H3 and HMC-1 

**Figure 5:**
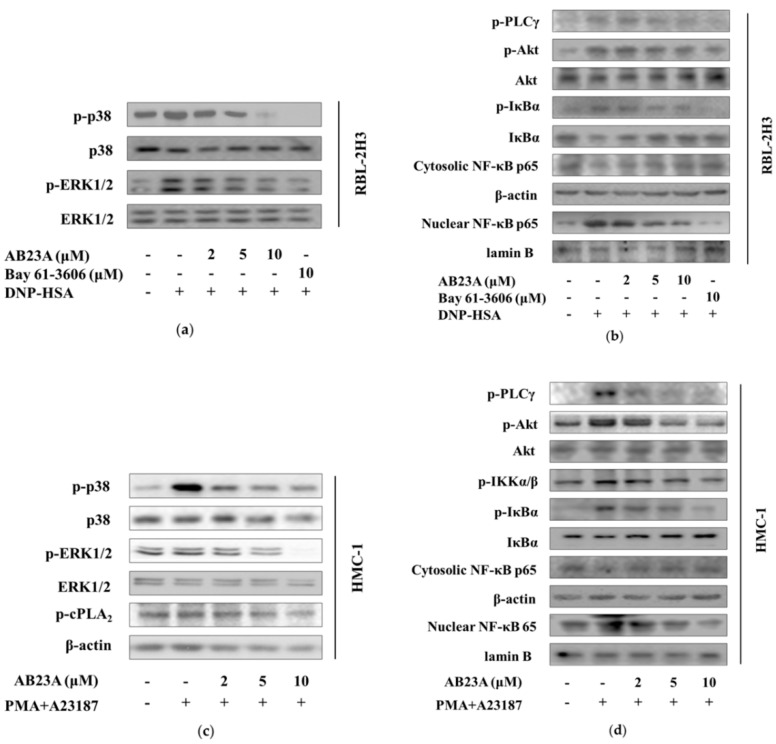
AB23A attenuates the phosphorylation of the IgE/Ag or phorbol 12-myristate 13-acetate (PMA)/A23187-stimulated signaling pathway in RBL-2H3 cells and HMC-1. (**a**,**b**) IgE-sensitized RBL-2H3 cells were pre-incubated with AB23A or Bay 61-3606 for 1 h, then stimulated with DNP-HSA for 15 min. Cell lysates were immunoblotted for p-p38, p-ERK1/2, p-PLCγ, p-Akt, p-IκBα, IκBα, and cytosolic and nuclear NF-κB p65. (**c**,**d**) HMC-1 cells were pre-treated with AB23A for 24 h, then stimulated with PMA plus A23187 for 20 min. Cell lysates were immunoblotted for p-p38, p-ERK1/2, p-cPLA2, p-PLCγ, p-Akt, p-IKKα/β, p-IκBα, IκBα, and cytosolic and nuclear NF-κB p65.

We also tested the effect of AB23A on other mast cells, RBL-2H3 and HMC-1. The concentrations of 2, 5, and 10 μM of AB23A didn’t significantly affect the cell viabilities in RBL-2H3 and HMC-1 (data not shown). As shown in Figure 5a, phosphorylation of p38 and ERK1/2 was increased by IgE/Ag-stimulation and these up-regulations were dose-dependently inhibited by AB23A. Meanwhile, AB23A reduced the phosphorylation of PLCγ and Akt, phosphorylation and degradation of IκBα, and translocation of NF-κB p65 from cytosol into the nucleus, see Figure 5b. 

Because HMC-1 cells lack expression of FcεRI, researchers use phorbol 12-myristate 13-acetate (PMA) plus calcium ionophore (A23187) to investigate the related mechanism. As shown in Figure 5c, under the stimulation of A23187 and PMA, elevated levels of phosphorylation of p38, ERK, and cPLA_2_ were inhibited by AB23A. Moreover, AB23A also attenuated the phosphorylation of PLCγ, Akt, p-IKKα/β, and phosphorylation and degradation of IκBα, and NF-κB p65 translocation induced by PMA plus A23187, see Figure 5d. 

### 2.6. AB23A Attenuates IgE-Mediated PCA

**Figure 6:**
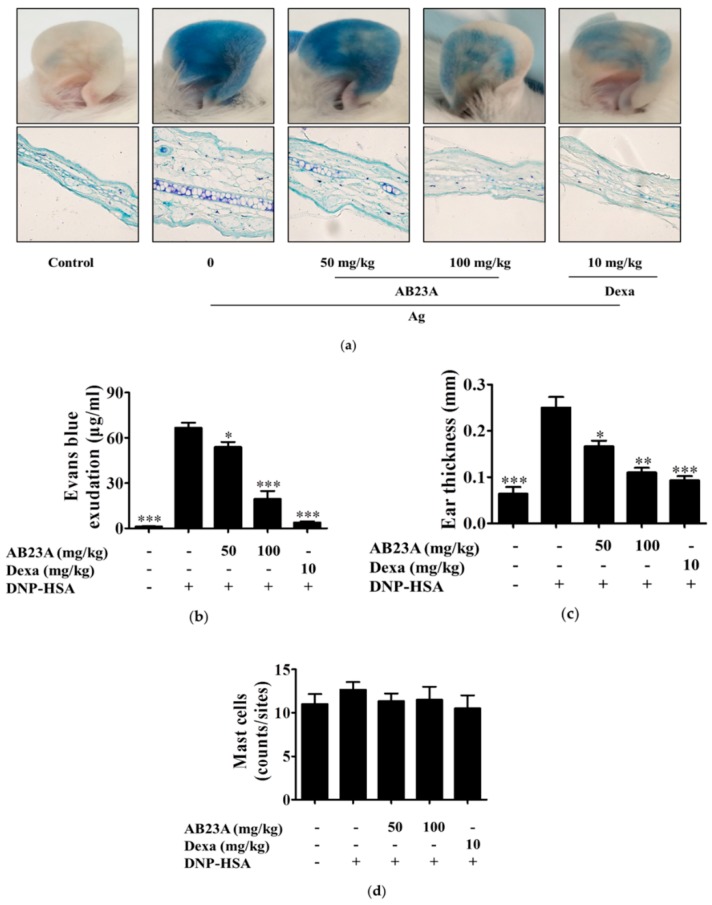
AB23A suppresses the IgE-mediated passive cutaneous anaphylaxis (PCA) reaction in mice. Institute of cancer research (ICR) mice were intradermally injected into both ears with 80 ng of anti-DNP IgE. After 24 h, AB23A or Dexa were orally treated 1 h before 60 μg of DNP-HSA containing 1% Evans blue was intravenously injected into the tails of the mice. (**a**) The representative images of ears and photomicrographs of ear sections were stained with toluidine blue as shown. (**b**) The dye extracted from the ear was detected using a spectrophotometer. (**c**) Ear thickness was measured. (**d**) The number of mast cells were counted at the dermis. The data show the mean ± SEM (*n* = 5 per group). ANOVA, *p* < 0.0001, post hoc * *p* < 0.05, ** *p* < 0.01 and *** *p* < 0.001, compared with the mice sensitized with anti-DNP IgE and challenged with DNP-HSA in the absence of AB23A.

Anaphylaxis is known to be mediated by IgE/Ag through cross-linking of FcεRI [20]. Mast cells play important roles in allergic response and anaphylaxis [20,21]. Therefore, we next investigated the anti-allergic effect of AB23A on the IgE-dependent PCA reaction in vivo. IgE was subcutaneously injected into mice on the ears, followed by oral administration of AB23A (50 and 100 mg/kg) or dexamethasone (Dexa) which was used as a positive drug. Then, DNP-HSA with 1% Evans blue dye was injected intravenously to induce PCA. As shown in Figure 6a, IgE-mediated PCA was successfully induced by the sequential injection of IgE and DNP-HSA in mice, and AB23A inhibited the allergic response in a dose-dependent manner, as reflected by the decrease in diffused dye and ear thickness with the AB23A treated group, see Figure 6b,c. Meanwhile, there was no significant change in the number of mast cells, as shown in Figure 6d. 

## 3. Discussion

Mast cells start to activate when allergen binds to a specific IgE antibody via the transmembrane protein of a high-affinity receptor (FcεRI) [22]. Upon activation, mast cells can release their granules such as histamine or generate several pro-inflammatory mediators such as LTC_4_, PGD_2_, IL-6, and TNF-α [23]. Lee et al. reported that AB23A inhibited LT production and β-hexosaminidase release in RBL-1 and RBL-2H3 cells, respectively [24]. However, the anti-allergic effect of AB23A in BMMC and the related mechanism have not yet been reported. In the present study, we demonstrated for the first time that AB23A diminished the degranulation in the IgE/Ag-stimulated BMMCs via inhibiting the FcεRI-mediated Syk signal pathway. Histamine has multiple physiological effects such as the increase of vascular permeability, smooth muscle contraction, and activation of nerves in immediate hypersensitivity reactions [9]. The release of histamine as the degranulation of mast cells is an important step in type I allergic reactions and immune responses. We found that AB23A impaired the release of histamine, see Figure 1c. Elevated Ca^2+^ mobilization causes degranulation, and phosphorylated PLCγ results in an increase of cytosolic Ca^2+^ concentration, and phosphorylated Syk can activate PLCγ. Our results showed that AB23A inhibited the mobilization of Ca^2+^ and the phosphorylation of PLCγ and Syk, see Figure 1d,e and Figure 4a. These data indicate that AB23A inhibits BMMCs degranulation by suppressing Syk and PLCγ phosphorylation and Ca^2+^ mobilization.

AB23A was also shown to inhibit LTC_4_ and IL-6 production and COX-2 expression, see Figure 2a and Figure 3a,b. LTs have been proven to be key mediators in asthma, allergic rhinitis, and other inflammatory conditions such as atopic dermatitis, urticaria, cardiovascular diseases, and cancer [12]. cPLA_2_ can be phosphorylated by MAPKs on Ser505 [25], and the activated cPLA_2_ preferentially hydrolyzes AA from the sn-2-position of membrane phospholipids in response to diverse cellular stimuli [14]. LTs are products of AA metabolism via the 5-LO pathway [26]. Here, we found that AB23A inhibits translocation of activated cPLA_2_ from cytosol to the nuclear envelope, see Figure 2b. Furthermore, AB23A impaired phosphorylation of p38 and ERK1/2, see Figure 2b. These results showed that AB23A inhibited LTC_4_ generation by suppressing the phosphorylation of p38 and ERK1/2 to prevent the translocation of cPLA_2_.

Transcription factor NF-κB dysregulation is associated with inflammatory diseases such as arthritis, asthma, and bowel disease. Therefore, NF-κB has been shown to be the target of several anti-inflammatory drugs [27,28]. PI3K-activated Akt activates IKK, and IKK complex phosphorylates the inhibitory IκBα proteins leading to their phosphorylation and ubiquitination. The released NF-κB translocates to the nucleus and transcripts various genes such as IL-6, TNF-α, and COX-2 [29,30]. In the present study, after the stimulation of BMMCs with IgE/Ag, Akt, and IKK were phosphorylated, IκBα was degraded, and NF-κB was activated, and these signal activations were significantly decreased by AB23A, see Figure 3c. Increases in cytosolic Ca^2+^ concentrations activate degranulation and also activate NF-κB which regulates the transcription of various cytokines [11]. Our result had shown that the intracellular level of Ca^2+^ was attenuated with AB23A treatment, see Figure 1d. Taken together, the suppression of IL-6 production and COX-2 expression by AB23A might be attributed to the inhibition of the Akt/IKK/NF-κB and Ca^2+^/NF-κB pathway.

Syk activation is central to mast cell effector function and allergic response [19]. IgE-mediated activation of mast cells is initiated through the aggregation of FcεRI by an antigen, with subsequent activation of the Lyn/Syk and Fyn, resulting in the activation of downstream signaling proteins [31,32]. AB23A downregulated phosphorylation of Syk, as well as downstream signaling proteins in BMMCs, but did not affect the activation of Lyn and Fyn, see Figure 4. Collectively, these findings suggest that AB23A can reduce allergic reactions by suppressing Syk-mediated signal molecules including MAPKs, NF-κB, and Ca^2+^. 

To confirm the anti-allergic mechanism of AB23A in mast cells, we used an RBL-2H3 cell, which is a mast cell line originating from rat basophilic leukemia and has been widely used to study IgE-FcεRI interactions. The results demonstrated that AB23A inhibited the activations of ERK, p38 MAPK, PLCγ, Akt, IκBα, and NF-κB, see Figure 5a,b, which is consistent with the results in IgE/Ag-stimulated BMMCs. Furthermore, we used human mast cell HMC-1 in which FcεRI is not expressed. Here, we found that AB23A also impaired the phosphorylation of p38 MAPK, ERK, PLA_2_, PLCγ, Akt, IKKα/β, phosphorylation and degradation of IκBα, and translocation of NF-κB into the nucleus, see Figure 5c,d, with a similar result to those for BMMCs and RBL-2H3. These data suggest that AB23A inhibits not only IgE/Ag-stimulated mast cells but also PMA plus A23187-stimulated mast cells. 

PCA is well known as a local allergic reaction mediated by antigen-stimulated mast cells in vivo, and the IgE-mediated mast cell activation is evaluated by vascular permeability increase [33]. Our study found that PCA was inhibited by the administration of AB23A in mice, whereas the number of mast cells in the allergic site at the ears was not changed, see Figure 6. These results suggest that AB23A may attenuate allergic responses by suppressing mast cell activation without altering the number of mast cells.

## 4. Materials and Methods 

### 4.1. Reagents

RPMI1640, fetal bovine serum (FBS), and the enhanced chemiluminescence (ECL) Western blot detection reagent were purchased from Thermo Fisher Scientific Inc. (Waltham, MA, USA). Mouse anti-dinitrophenyl (DNP) IgE was purchased from Sigma Chemicals (St. Louis, MO, USA). DNP-HSA was from Biosearch Technologies (Petaluma, CA, USA). The antibodies specific for phospho-ERK1/2, ERK1/2, phospho-p38, p38, phospho-PLCγ, phospho-IκB, IκB, phospho-IKKα/β, β-actin, and the horseradish peroxidase-conjugated goat anti-rabbit secondary antibody were purchased from Cell Signaling Technology, Inc. (Danvers, MA, USA). The antibodies specific for phospho-cPLA_2_, NF-κB p65, lamin B, Lyn, Fyn, and Syk, as well as Bay 61-3606 reagent were obtained from Santa Cruz Biotechnology, Inc. (Dallas, TX, USA). The LTC_4_ enzyme linked immunoassay (EIA) kit, and the antibody for COX-2 were from Cayman Chemical (Ann Arbor, MI, USA). Histamine enzyme linked immunosorbent assay (ELISA) kit was purchased from Demeditec Diagnostics GmbH (Kiel, Germany). AB23A was obtained from Push Bio-technology Co., Ltd (Chengdu, China), of which the purity is ≥98%.

### 4.2. Cell Culture and Activation

BMMCs were isolated from the bone marrow of Balb/c mice and differentiated as described previously by us [34]. RBL-2H3 and HMC-1 were cultured without IL-3. BMMCs and RBL-2H3 were sensitized with 500 ng/mL of anti-dinitrophenyl (DNP) IgE overnight and then treated with AB23A or Bay 61-3606 for 1 h. After being stimulated with 100 ng/mL of DNP-HSA (used as Ag) for 15 min or 6 h, the supernatants were collected for further analysis, respectively. The levels of LTC_4_, histamine and IL-6 were determined using ELISA according to the manufacturer’s protocol. The HMC-1 cells were incubated with AB23A in the presence or absence of calcium ionophore (A23187) and phorbol 12-myristate 13-acetate (PMA) for further analysis.

### 4.3. Cell Viability

Cell viability was assessed using an MTT assay. Briefly, BMMCs were seeded onto a 96-well culture plate and incubated with various concentrations of AB23A for 8 h. A total of 20 μL of MTT (5 mg/ml) was added to each well; RBL-2H3 and HMC-1 cells were seeded onto a 96-well culture plate and incubated with various concentrations of AB23A for 24 h, then MTT was added to each well. After 4 h incubation, the formazan was dissolved in DMSO, and optical density (OD) at 490 nm was measured using microplate reader iMark (BIO-RAD, Hercules, CA, USA).

### 4.4. Measurement of Intracellular Ca^2+^ Level

The intracellular Ca^2+^ level was determined with the FluoForte Calcium Assay Kit (Enzo Life Sciences, Ann Arbor, MI, USA), as described previously [34]. Briefly, IgE-sensitized BMMCs were pre-incubated with FluoForte^TM^ dye-loading solution for 1 h, and then treated with AB23A or Bay 61-3606 for 1 h. After addition of DNP-HSA and incubation for 5 min, the fluorescence was monitored with a multilabel plate reader at Ex = 485 nm/Em 535 nm (Perkin Elmer’s VICTORTMX5 Multilabel Plate Reader, Waltham, MA, USA).

### 4.5. Extraction of Nuclear Proteins

IgE-sensitized BMMCs and RBL-2H3 cells were pretreated with AB23A or Bay 61-3606 for 1 h, followed by incubation with DNP-HSA for 30 min; HMC-1 cells were pretreated with AB23A for 1 h and incubated with PMA plus A23187 for 20 min. Then, the nuclear proteins were prepared by using Nuclear Extraction Kit according to the manufacturer’s protocol (Panomics, Fremont, CA, USA).

### 4.6. Western Blot Analysis

IgE-sensitized BMMCs or RBL-2H3 were pretreated with AB23A or Bay 61-3606 for 1 h and stimulated with DNP-HSA. To assess COX-2 expression, IgE-sensitized BMMCs were pre-incubated with 1 μg/ml aspirin for 2 h to eliminate preexisting COX-1. After washing, the BMMCs were stimulated with DNP-HSA in the presence or absence AB23A for 7 h. For HMC-1, cells were pretreated with AB23A for 1 h stimulated with PMA plus A23187 for 20 min, then collected to prepare the cell lysates for follow-up experiments. Western blot analysis was carried out as we reported previously [35]. Equal amounts of protein in the cell lysates were separated by sodium dodecyl sulfate-polyacrylamide gel electrophoresis (SDS-PAGE) and transferred onto a PVDF membrane (Millipore, Billerica, MA, USA). After blocking in 5% non-fat milk, the membranes were incubated with specified primary antibodies and then the respective secondary antibodies. Immunoreactive bands were visualized using enhanced ECL reagents and quantified using Image J software. The signals were quantified by comparison to that of β-actin (for total or cytosolic protein) or lamin B (for nuclear protein) band. 

### 4.7. Immunoprecipitation (IP)

IP was performed as we described previously [36]. Total cell lysates were incubated with anti-Syk, anti-Lyn or anti-Fyn antibodies overnight at 4 °C, and immune-complexes were precipitated with protein A/G plus agarose. These precipitates were then washed four times with ice-cold lysis buffer, subjected to SDS-PAGE, and immunoblotted with the respective antibodies.

### 4.8. IgE-Mediated PCA in Mice

ICR mice (7 weeks old, male) were subcutaneously injected with 80 ng of anti-DNP IgE (Sigma) into each ear, 24 h before the Ag challenge. After IgE-sensitization, the mice were orally administered AB23A or Dexa. One hour later, the mice were intravenously challenged with 60 µg of DNP-HSA in 200 μL of PBS containing 1% (*w*/*v*) Evans blue. After 1 h, mice were euthanized and the ears were removed. The ear tissue absorbed dye was extracted with 400 μL of formamide at 63 °C overnight and the absorbance was measured at 630 nm using a microplate reader (BIO-RAD iMark, Hercules, CA, USA). The ears were fixed with 4% formaldehyde and embedded in paraffin. Five μm sections of the tissues were prepared and stained with toluidine blue to count the number of mast cells and calculate ear thickness. Procedures used in this study were approved by the Institutional Animal Care and Use Committee of Tianjin Medical University (project identification code TMUaMEC2017022, 5 November 2017).

### 4.9. Statistical Analysis

All values are expressed as means ± SEM of triplicate values. One-way ANOVA followed by Tukey's Multiple Comparison Test was utilized to determine the statistical significance with GraphPad Prism 5 (GraphPad, San Diego, CA, USA). Differences were considered statistically significant when *p* < 0.05.

## 5. Conclusions

**Figure 7:**
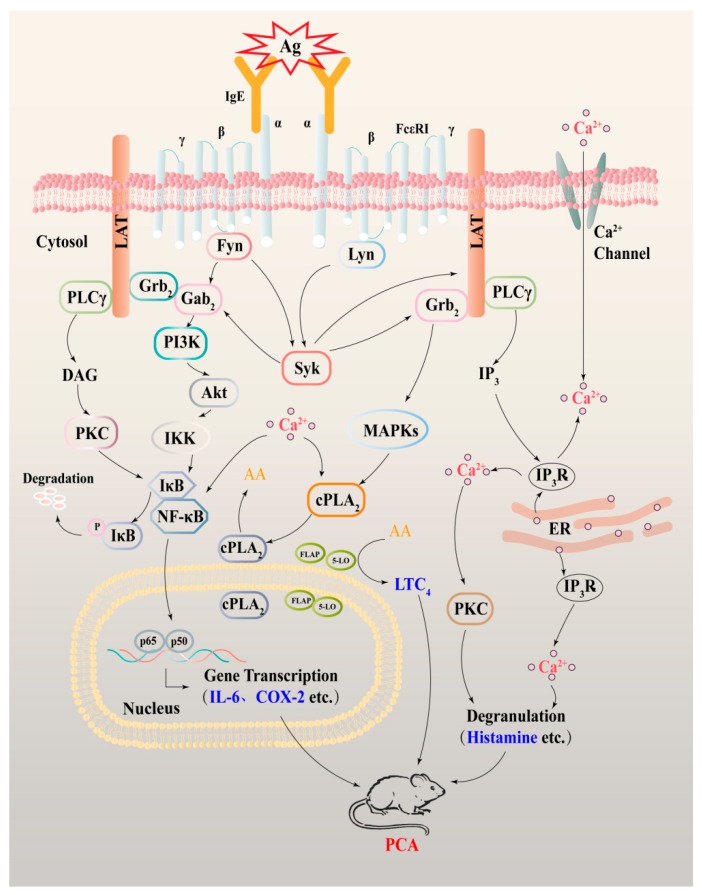
Schematic diagram describing the effect of AB23A on IgE/Ag-mediated mast cell activation and allergic PCA reaction.

In conclusion, the present study demonstrated that AB23A has anti-allergic effects in IgE/Ag-activated BMMCs and RBL-2H3 and PMA/A23187-activated HMC-1 cells. The mechanisms responsible for the anti-allergic effects may involve Syk-mediated down-stream signals including PLCγ, ERK, and p38 MAPK, NF-κB, cPLA_2_, COX-2, and Ca^2+^ release, see Figure 7. Furthermore, AB23A could attenuate the allergy response of the PCA reaction in vivo, via suppressing the vascular permeability and ear swelling response. AB23A might become a lead compound in the treatment of allergic inflammatory diseases.

## Abbreviations

AB23A	Alisol B 23-acetate
IgE	Immunoglobulin E
FcεRI	Immunoglobulin E receptor
LTC_4_	Leukotriene C_4_
IL-6	Interlukin-6
COX-2	Cyclooxygenase-2
BMMCs	Bone marrow-derived mast cells
RBL-2H3	Rat basophilic leukemia
HMC-1	Human mast cell line
Syk	Spleen tyrosine kinase
PLCγ	Phospholipase Cγ
MAPKs	Mitogen-activated protein kinases
cPLA_2_	Cytosolic phospholipase A_2_
PMA	Phorbol 12-myristate 13-acetate
A23187	Calcium ionophore
PCA	Passive cutaneous anaphylaxis
